# MB2033, an anti-PD-L1 × IL-2 variant fusion protein, demonstrates robust anti-tumor efficacy with minimal peripheral toxicity

**DOI:** 10.1007/s00262-024-03742-1

**Published:** 2024-06-04

**Authors:** Young Jin Park, Suna Kim, Hyoju Bang, Seok Chan Kang, Sunjung Cho, Jun-Eui Park, Sungyoub Jung, Ha Hyung Kim

**Affiliations:** 1 Research center, Mustbio, 102 Edu town-ro, Yeongtong-gu, Suwon-si, Gyeonggi-do 16509 Republic of Korea; 2 Department of Global Innovative Drugs, Graduate School of Chung-Ang University, 84 Heukseok-ro, Dongjak-gu, Seoul, 06974 Republic of Korea

**Keywords:** Interleukin-2, Immune checkpoint inhibitor, Immunotherapy, Bispecific fusion protein

## Abstract

Interleukin-2 (IL-2), a cytokine with pleiotropic immune effects, was the first approved cancer immunotherapy agent. However, IL-2 is associated with systemic toxicity due to binding with its ligand IL-2Rα, such as vascular leakage syndrome, limiting its clinical applications. Despite efforts to extend the half-life of IL-2 and abolish IL-2Rα interactions, the risk of toxicity remains unresolved. In this study, we developed the bispecific fusion protein MB2033, comprising a novel IL-2 variant (IL-2v) connected to anti-programmed death ligand 1 (PD-L1) via a silenced Fc domain. The IL-2v of MB2033 exhibits attenuated affinity for IL-2Rβγ without binding to IL-2Rα. The binding affinity of MB2033 for PD-L1 is greater than that for IL-2Rβγ, indicating its preferential targeting of PD-L1^+^ tumor cells to induce tumor-specific immune activation. Accordingly, MB2033 exhibited significantly reduced regulatory T cell activation, while inducing comparable CD8^+^ T cell activation to recombinant human IL-2 (rhIL-2). MB2033 induced lower immune cell expansion and reduced cytokine levels compared with rhIL-2 in human peripheral blood mononuclear cells, indicating a decreased risk of peripheral toxicity. MB2033 exhibited superior anti-tumor efficacy, including tumor growth inhibition and complete responses, compared with avelumab monotherapy in an MC38 syngeneic mouse model. In normal mice, MB2033 was safer than non-α IL-2v and tolerable up to 30 mg/kg. These preclinical results provide evidence of the dual advantages of MB2033 with an enhanced safety and potent clinical efficacy for cancer treatment.

**Supplementary Information:**

The online version contains supplementary material available at 10.1007/s00262-024-03742-1.

## Introduction

Programmed death ligand 1 (PD-L1) is a critical immune checkpoint protein, representing an important target in cancer immunotherapy, as the overexpression of PD-L1 on the surface of tumor cells is a common immune evasion strategy of cancer cells [1]. The mechanism of tumor immunosuppression by the PD-1/PD-L1 pathway mainly involves the activation of T cells to facilitate the immune tolerance of tumor cells, allowing them to evade the immune system [2]. Accordingly, immune checkpoint inhibitors (ICIs) targeting the PD-1/PD-L1 interaction have emerged as a revolutionary approach in cancer immunotherapy. Blockade of the PD-1/PD-L1 interaction reinvigorates the activity of cytotoxic T cells, enhancing their ability to recognize and eliminate cancer cells, ultimately resulting in a potent anti-tumor immune response. To date, ICI therapy has shown promising results for various cancer types, including melanoma and non-small cell lung carcinoma [3–5]. Although ICIs are considered an innovative therapeutic approach in immuno-oncology, clinical limitations remain to be overcome, including lack of efficacy in some cases, low overall response rates, development of resistance, and side effects [6].

Interleukin (IL)-2 is a pioneering immunomodulatory cytokine and potent immunotherapeutic agent [7]. IL-2 exhibits pleiotropic effects, controlling immune-inflammatory responses by engaging effector T, natural killer (NK), and B cells, while restraining autoimmune reactions via regulatory T cells (Tregs). These contrasting roles are enabled by the distinct expression of three IL-2 receptor (IL-2R) subunits, namely IL-2Rα, β, and γ, on effector cells, along with the varying affinity of IL-2 for specific receptor configurations. Inactive NK and CD8^+^ T cells exclusively express the intermediate-affinity IL-2 receptor IL-2Rβγ, whereas Tregs express the high-affinity receptor IL-2Rαβγ [8]. Although IL-2Rα is constitutively expressed on Tregs, its expression can be induced in CD8^+^ effector T cells and NK cells in response to activation [9].

Despite its potential therapeutic benefits, high-dose IL-2 therapy is rarely used in clinical practice because of severe adverse effects in many patients, including fever, malaise, and vascular leakage syndrome [10]. Consequently, approximately 50% of patients discontinue therapy, and 2–5% of patients experience therapy-related edema and vascular instability, leading to death [11]. The systemic adverse effects of IL-2 therapy are primarily caused by the potent off-target binding to IL-2R. The high affinity for the IL-2Rα of wild-type (WT) IL-2 drives the expansion of immunosuppressive Tregs. Similarly, the robust affinity for IL-2βγ prompts excessive activation of abundant NK cells, potentially inducing unexpected immune reactions.

These challenges have spurred focused exploration into refining the therapeutic potential of IL-2 while mitigating its limitations. To date, researchers have primarily focused on suppressing its binding to IL-2Rα to prevent Treg activation and enhance the therapeutic efficacy. For example, bempegaldesleukin was once considered a promising IL-2-based therapy [12]. Despite efforts to mitigate toxicity by modifying the structure of IL-2, bempegaldesleukin, designed to selectively target IL-2Rβγ, failed to demonstrate significant improvement over conventional ICIs in clinical trials, leading to its discontinuation in phase 3 studies [13]. These challenges underscore the need to develop safer and more effective IL-2-based alternatives. As potential game-changers, albeit with some limitations, nemvaleukin alfa [14] and SAR444245 [15] were developed as a new class of non-α IL-2 agents, representing the latest generation of candidate IL-2-based therapies. Although these novel drugs have the potential to reduce the toxicity of IL-2, when administered as monotherapy, they exhibit very low efficacy and can be used at low doses to avoid the induction of toxicity.

The synergy of PD-L1 targeting and IL-2 immunostimulation presents a promising strategy for cancer immunotherapy, marking a significant breakthrough in oncology. This dual approach enables tumor-specific targeting, while avoiding the induction of systemic inflammation caused by cytokines, such as cytokine release syndrome and vascular leakage, through the off-target effects of IL-2 on peripheral effector and endothelial cells. Although non-α IL-2 variants (IL-2vs) may be safer than WT IL-2 and improve efficacy by inducing less Treg activation, concerns persist regarding potential overactivation of IL-2Rβγ-expressing cells, possibly leading to NK cell-driven toxicity [16, 17]. Therefore, there is a need to develop IL-2v drugs that are safer and more effective than non-α IL-2.

We developed a strategy to enhance the anti-tumor efficacy of IL-2 therapy by selectively enhancing the expansion and activation of CD8^+^ T cells over Tregs in tumors with abundant PD-L1 expression. Therefore, we constructed the novel variant fusion protein MB2033 comprising anti-human PD-L1 antibody and low-affinity IL-2v, and its anti-tumor efficacy was assessed in vitro and in vivo.

## Materials and methods

### Cell culture

Human breast cancer MDA-MB-231 cells (ATCC, Manassas, VA, USA) were cultured in RPMI-1640 supplemented with 10% fetal bovine serum (FBS) and 1% penicillin/streptomycin (Gibco, Carlsbad, CA, USA). Human embryonic kidney HEK293T cells (ATCC) were cultured in Dulbecco’s modified Eagle medium supplemented with 10% FBS and 1% penicillin/streptomycin (Gibco).

### Generation and purification of the bispecific fusion protein

The MB2033 construct was engineered by incorporating an anti-PD-L1 fragment antigen-binding region (Fab), a novel IL-2v, and heterodimeric IgG1 Fc to avoid non-specific and systemic FcγR-mediated responses, using BICSTA™ (Best-In-Class in multi-Specific Targeting), a unique technology that allows the highly efficient heterodimerization of Fcs. Anti-PD-L1 Fab was linked to Fc1 by a hinge, and the novel IL-2v was linked to Fc2 by a (G_4_S)_3_ linker. The gene encoding MB2033 was synthesized using GeneArt AG technology (Invitrogen; Thermo Fisher Scientific, Waltham, MA, USA). The Fc1 and Fc2 constructs were cloned into the pCHO1.0 vector separately. The Fc1 and Fc2 expression vectors were then mixed and transfected into the ExpiCHO-S™ cell line using the ExpiFectamine™ CHO transfection kit (Thermo Fisher Scientific). After culturing the transfected cells, the supernatant was harvested and purified using MabSelect Prism A and Source30S columns (Cytiva, Marlborough, MA, USA).

The non-α IL-2v-Fc (no binding to IL-2Rα), non-α and βγ-attenuated IL-2-Fc (MB2033), αPD-L1 × non-α IL-2v, and αPD-L1 × WT IL-2 constructs were cloned into the pCHO1.0 vector and expressed by transient transfection of Expi-CHO-S™ cells. After culturing, the supernatant was purified using MabSelect Prism A and/or Source30S columns.

### Surface plasmon resonance (SPR) analysis

The binding affinity of MB2033 for hPD-L1 and IL-2R was evaluated using SPR with a Biacore T200 system (Cytiva). In brief, hPD-L1, hIL-2Rα, hIL-2Rβ, hIL-2Rβγ, or IL-2Rαβγ (Acrobiosystems, Newark, DE, USA) were immobilized on a CM5 sensor chip. A dilution series of test articles was injected over the surface, and the binding kinetics were monitored. The binding affinity (*K*_*D*_) was calculated using the association (*k*_a_) and dissociation (*k*_d_) constants.

### PD-1/PD-L1 competitive enzyme-linked immunosorbent assay (ELISA)

To compare the blockade function of MB2033 with avelumab, the PD-1/PD-L1 interaction was observed using the hPD-1/hPD-L1 Inhibitor Screening Assay Kit (BPS Bioscience, San Diego, CA, USA). Chemiluminescence was measured using a Varioskan LUX Multimode Microplate Reader (Thermo Fisher Scientific).

### Analysis of binding properties to PD-L1^+^ tumor cells

MDA-MB-231 or HEK293T cells were washed with staining buffer (BD Biosciences, San Jose, CA, USA) and incubated with avelumab (positive control) or MB2033 at various concentrations for 30 min. After washing again with the staining buffer, the cells were incubated with anti-human IgG conjugated with BV421 (BD Biosciences). Live cells were analyzed using a flow cytometer (BD FACSLyric™; BD Biosciences) to assess the binding levels with avelumab or MB2033.

### Phosphorylation of signal transducer and activator of transcription 5 (pSTAT5) assay

To discover an appropriate IL-2v with both efficacy and safety, pSTAT5 assay was conducted to evaluate the level of immune cell activation following treatment with IL-2 drugs. Human peripheral blood mononuclear cells (hPBMCs; StemCell Technologies, Cambridge, MA, USA) were stained with CD3-BV605, CD4-FITC, CD8-APC-H7, and CD25-BV480. The hPBMCs were then stimulated with the test agents for 20 min at 37 °C, immediately fixed with Transcription Factor Buffer Set and additionally permeabilized with perm III buffer. After intracellular staining with pSTAT5-Alexa647 and FoxP3-PE for 30 min, changes in the levels of pSTAT5 on CD8^+^ T and Treg cells were measured by flow cytometry.

### hPBMC expansion assay in vitro

To demonstrate the potency of CD8^+^ T cell proliferation in cell populations, compared the expansion levels of immune cells in response to each drug. hPBMCs were cultured with either 0.5 nM (EC_50_ value observed for MB2033 pSTAT5 results) aldesleukin (positive control), αPD-L1 × IL-2 (WT control), αPD-L1 × non-α IL-2v, or MB2033 and 0.3 μg/mL anti-CD3 (BioLegend, San Diego, CA, USA) for the activated condition or without anti-CD3 for the resting condition. On days 3 and 5, fresh medium containing the test compounds was added to the culture flask or scaled up. Total cells were collected on day 7, counted, stained with fluorescent-conjugated antibodies, and subjected to flow cytometry (BD Biosciences) to determine the proportions of CD8^+^ T cells, NK cells, and Tregs among the total lymphocytes.

### Cytotoxicity analysis

To determine the tumor-killing effect conferred by the increased immune response induced by MB2033 at a concentration corresponding to the EC_90_ within effective concentrations, Hoechst 33342 (Thermo Fisher Scientific)-stained MDA-MB-231 breast cancer cells were co-cultured with Cell Tracker Orange-labeled hPBMCs at an effector-to-target ratio of 50:1. The test drugs were administered followed by staining for caspase 3/7, an apoptosis marker, with Early Tox dye (Molecular Devices, San Jose, CA, USA). During incubation for 48 h, live cell images were captured at 2-h intervals using the Xpress live cell imaging system (Molecular Devices). The overlapping area of MDA-MB-231 (blue color) and hPBMCs (orange color) was designated as the “cancer clump (white)”, and the intensity of the caspase 3/7^+^ signal (green color) was measured within the cancer clump.

### Cytokines analysis

hPBMCs were incubated in the presence of aldesleukin, αPD-L1 × non-α IL-2v, or MB2033 at 10 or 100 nM (concentrations above the EC_90_ to confirm safety) for 72 h. The levels of interferon (IFN)-γ, IL-6, and tumor necrosis factor-alpha (TNF-α) in cultured hPBMCs were simultaneously measured using a BD Cytometric Bead Array (CBA; BD Biosciences).

### In vivo therapeutic effects in a syngeneic mouse model

The animal study was reviewed and approved by the Institutional Animal Care and Use Committee of Ahn-Gook Pharmaceutical (approval number: AG-IACUC-2022–06). C57BL/6 mice (6–8 weeks; female) were purchased from Koatech (Korea) and housed in an animal facility under controlled conditions: a temperature of (23 ± 3 °C), relative humidity of (55 ± 15%), and a 12-h light–dark cycle. For establishment of a colon cancer syngeneic model, the mice were subcutaneously injected with 1 × 10^6^ MC38 colon cancer cells in their right flanks. The mice were randomized into each group with an average tumor volume of 50–150 mm^3^: One group was intraperitoneally administered avelumab (10 mg/kg) once a week for 2 weeks, and the other groups were administered MB2033 (16 mg/kg) as a single injection or once a week for 2 weeks. Tumor volume was measured three times a week using calipers.

### Toxicity evaluation in normal mice

This animal study was reviewed and approved by the Institutional Animal Care and Use Committee of Chaon Co. Ltd. (approval number: CE23320). To compare the toxicity according to the binding affinity for IL-2Rs, female C57BL/6 mice aged 7 weeks (Nara Biotech, Korea) were intraperitoneally injected with non-α IL-2v-Fc (3 or 10 mg/kg, *n* = 5 each), IL-2v (non-α and βγ-attenuated; MB2033)-Fc (3, 10, or 30 mg/kg; *n* = 5 each), αPD-L1 × non-α IL-2v (10 or 30 mg/kg; *n* = 3 each), or MB2033 (10 or 30 mg/kg; *n* = 5 each). Body weights and general clinical signs were recorded daily.

### Mouse pharmacokinetics

This animal study protocol was reviewed and approved by the Institutional Animal Care and Use Committee of Keyfronbio Co., Ltd., based on the Animal Protection Act of the Republic of Korea (approval number: KA23104). Blood samples were collected from male ICR mice (Orientbio Inc., Korea) following a single intravenous administration of MB2033 (1, 3, or 10 mg/kg; *n* = 3/group) or aldesleukin (1 mg/kg). Serum concentrations of MB2033 were quantified using an in-house sandwich ELISA method. The concentration of aldesleukin in the collected mouse serum was measured using a commercial ELISA kit (R&D Systems Inc., Minneapolis, MN, USA). The pharmacokinetic parameters were calculated using a non-compartmental analysis program with Phoenix WinNonlin 8.3.4.295 software (Certara).

### Statistical analysis

All data were analyzed using GraphPad Prism 10 (La Jolla, CA, USA) with Student’s t test or one-way ANOVA. The level of significance was set at *p* < 0.05.

## Results

### MB2033 selectively delivers IL-2v with attenuated IL-2R interactions to PD-L1-expressing tumor cells

**Fig. 1:**
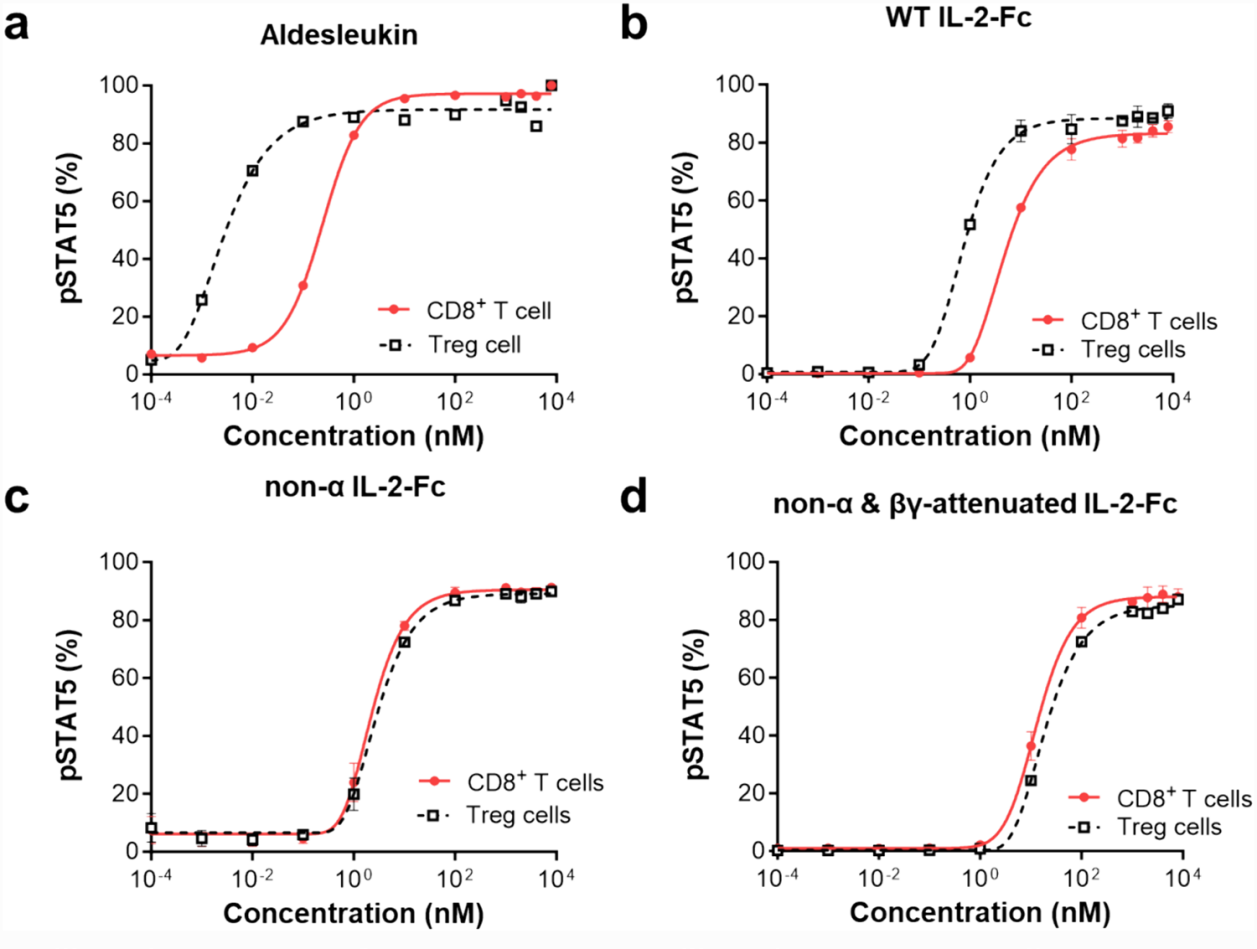
Non-α and attenuated βγ IL-2 selectively triggers STAT5 signaling in CD8^+^ T cells rather than Treg cells compared with wild-type IL-2

Aldesleukin and WT IL-2-Fc induced greater activation of Tregs than of CD8^+^ T cells (Fig. 1a, b). However, both non-α-IL-2-Fc and non-α and βγ-attenuated IL-2-Fc induced pSTAT5 in CD8^+^ T cells and Tregs to the same degree (Fig. 1c, d). These results indicated that non-α and non-α and βγ-attenuated IL-2vs could avoid off-target side effects by reducing the activation and expansion of Tregs compared to CD8^+^ T cells.

hPBMCs were incubated with increasing concentrations of **a** aldesleukin, **b** WT IL-2-Fc, **c** non-α IL-2-Fc, and **d** non-α and βγ-attenuated IL-2-Fc for 20 min and were analyzed by flow cytometry for percent of intracellular phosphorylated-STAT5^+^ cells in CD8^+^ T cells or Treg cells. The results are representative of at least two independent experiments. Data are shown as the means ± SEMs of duplicates.

**Fig. 2:**
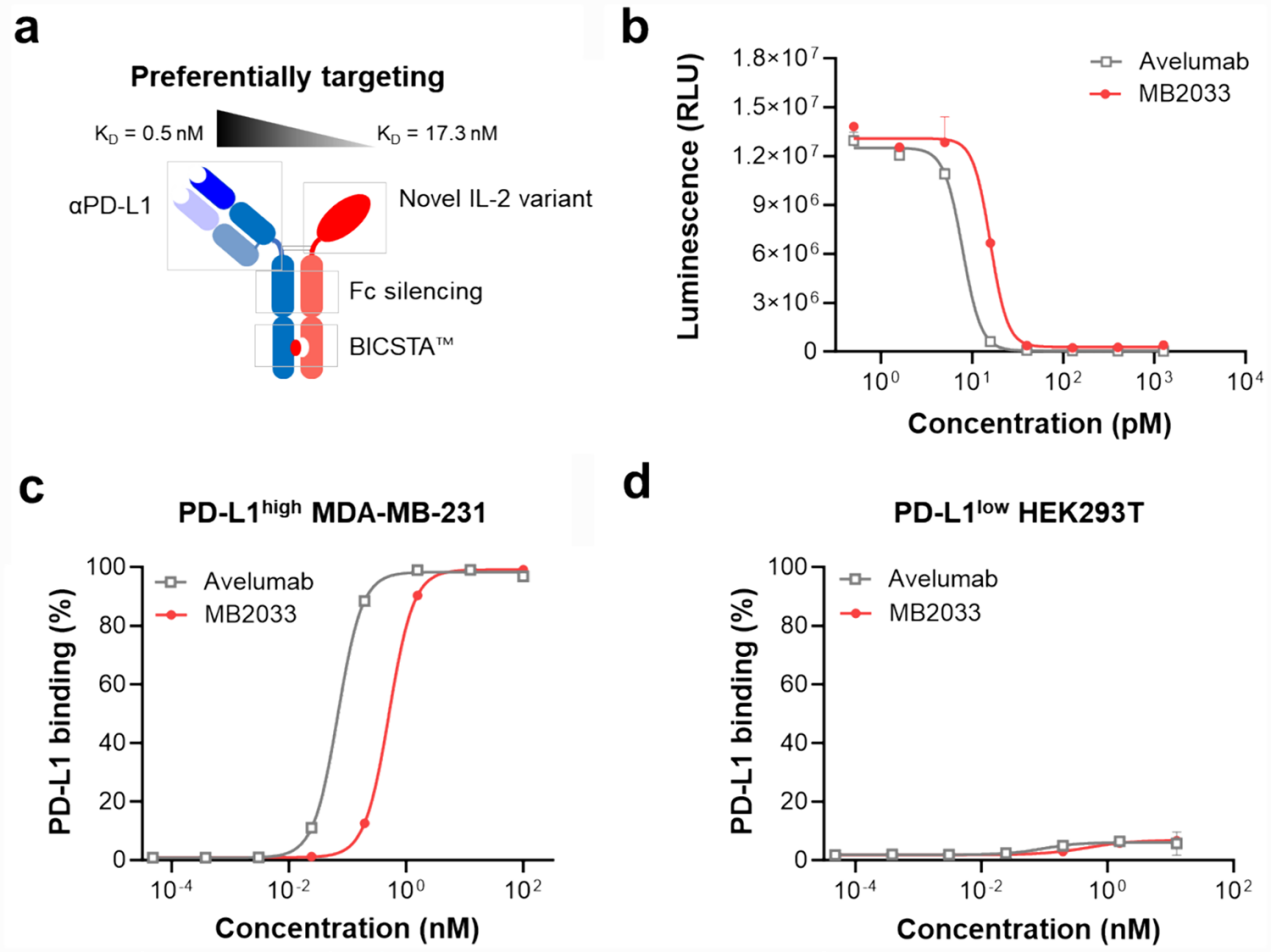
MB2033 preferentially targets to tumor cells expressing high levels of PD-L1. **a** Schematic representation and its features of MB2033. MB2033 is designed to preferentially target PD-L1 and binds to IL-2Rβγ without binding to IL-2Rα. Binding affinity (*K*_*D*_) of MB2033 to PD-L1 (left arm) and IL-2Rαβγ (right arm) was measured by SPR analysis. **b** Inhibitory curves for MB2033 of the PD-1/PD-L1 interaction relative to the avelumab were evaluated using the inhibitor screening assay. **c, d** Binding characteristics of MB2033 on PD-L1-expressed cell. Human MDA-MB-231 (PD-L1-highly expressed cells) and HEK293T (PD-L1-lowly expressed cells) were treated with MB2033 or avelumab and analyzed by flow cytometry. The results are representative of at least three independent experiments

To direct IL-2v delivery to target tumor cells, we engineered a fusion protein (MB2033) that combines an anti-PD-L1 Fab with a novel IL-2v. We confirmed that the novel IL-2v lacks binding affinity to IL-2Rα and exhibits low affinity for IL-2Rαβγ (*K*_*D*_ = 17.3 nM), along with biased affinity toward hPD-L1 (*K*_*D*_ = 0.5 nM) over IL-2Rαβγ by approximately 34.6-fold (Fig. 2a, Table 1). Based on the SPR results, we expected that MB2033 would demonstrate greater functional affinity for hPD-L1 expressed on tumor cells relative to its affinity for IL-2Rs on immune cells when compared with WT IL-2-based immune cytokines (Fig. 2b). Consistent with the SPR results, the inhibitor screening assay showed that MB2033 (half-maximal effective concentration [EC_50_] = 15.8 nM) interfered with the PD-1/PD-L1 interaction at a level approximately two-fold lower than that of avelumab (EC_50_ = 7.8 nM), which was attributed to the difference between the monovalent and bivalent antibodies. To examine the hPD-L1-dependent binding of MB2033, we performed a cell-based binding analysis using PD-L1^high^ MDA-MB-231 and PD-L1^low^ HEK293T cells. PD-L1^high^ and PD-L1^low^ cells were differentiated based on the flow cytometry (Supplementary Fig. 2). MB2033 efficiently bound to hPD-L1 high-expressing MDA-MB-231 cells (Fig. 2c) but not to hPD-L1 low-expressing HEK293T cells (Fig. 2d), confirming that MB2033 can induce a response dependent on the expression of PD-L1 on cells and would have tumor-targeting function.

**Table 1:** The binding affinity of MB2033 to PD-L1 and IL-2Rs determined by SPR analysis

Ligand	Test article	Binding affinity (*K*_*D*_, nM)
hPD-L1	Avelumab	0.2
	MB2033	0.5
hIL-2Rα	Aldesleukin	35.7
	MB2033	No binding
hIL-2Rβ	Aldesleukin	775
	MB2033	2828
hIL-2Rβγ	Aldesleukin	1.6
	MB2033	37.9
hIL-2Rαβγ	Aldesleukin	0.1
	MB2033	17.3

### MB2033 triggers the functional IL-2 signaling pathway and promotes expansion of CD8^+^ T cells rather than Tregs

**Fig. 3:**
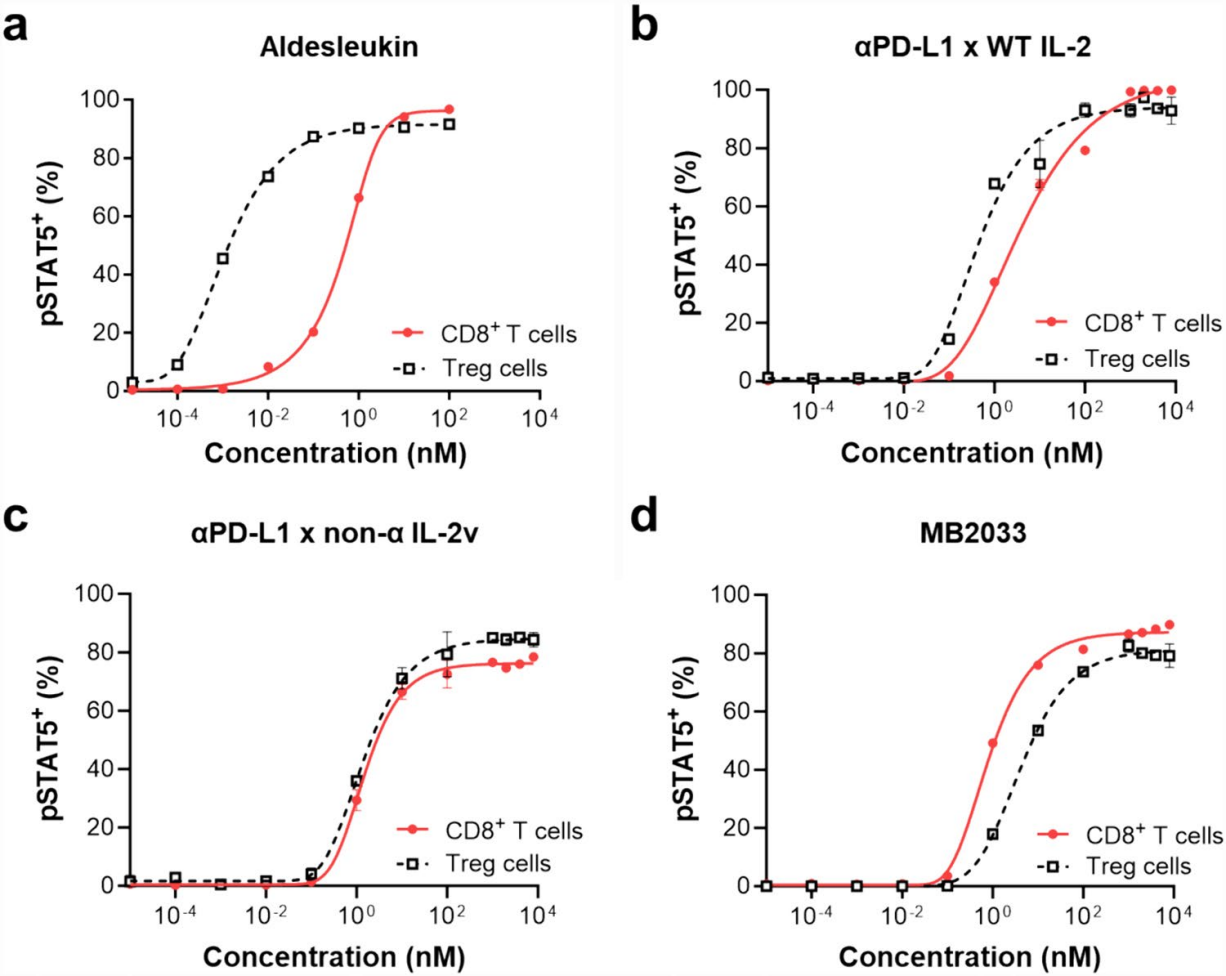
MB2033 induces IL-2R signaling, resulting in pSTAT5 in CD8^+^ T cells in vitro. hPBMCs were incubated with increasing concentrations of **a** aldesleukin, **b** αPD-L1 × WT IL-2, **c** αPD-L1 × non-α IL-2v, and **d** MB2033 for 20 min and were analyzed by flow cytometry for percent of intracellular pSTAT5^+^ cells in CD8^+^ T cells or Treg cells. The results are representative of at least three independent experiments. Data are shown as the means ± SEMs of duplicates

Aldesleukin, WT IL-2, enhanced STAT5 phosphorylation in Tregs more than that in CD8^+^ T cells (Fig. 3a). This result is consistent with the fact that Tregs express the high-affinity IL-2Rs, characterized by IL-2Rαβγ, further supporting the need to reduce the affinity of IL-2 for IL-2Rα to increase the immune-activating response in tumors. Likewise, the MB2033 analog with WT IL-2 (αPD-L1 × WT IL-2) also induced pSTAT5 preferentially in Tregs over CD8^+^ T cells (Fig. 3b). However, the αPD-L1 × non-α-IL-2v construct, including an IL-2v that does not bind to IL-2Rα, exhibited a tendency to induce similar levels of activation for Tregs and CD8^+^ T cells (Fig. 3c). MB2033, comprising an IL-2v that does not bind to IL-2Rα and has attenuated binding to IL-2Rβγ, showed the selective activation of CD8^+^ T cells over Tregs (Fig. 3d), demonstrating its potential to further induce the selective proliferation of CD8^+^ T cells.

**Fig. 4:**
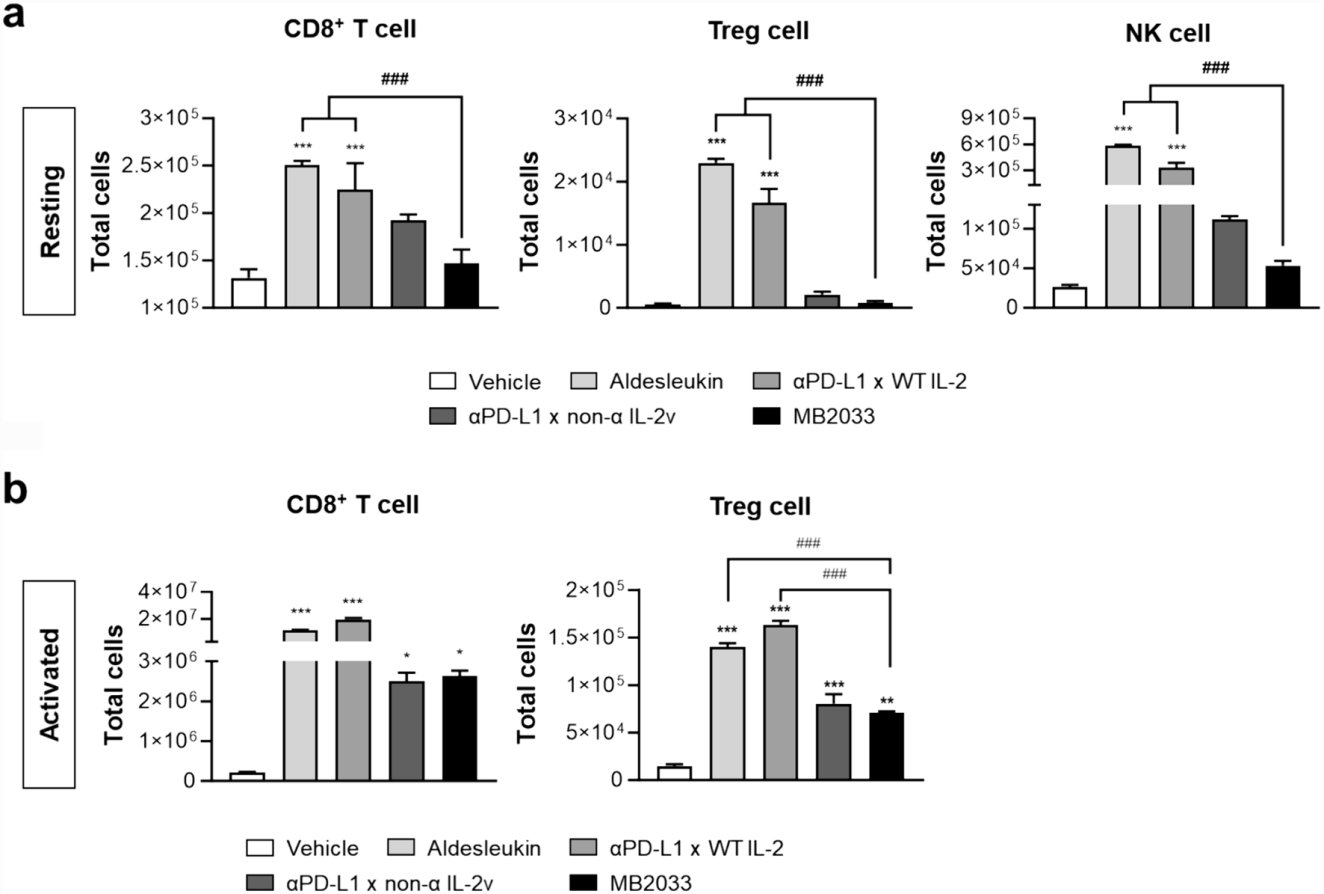
MB2033 induced the expansion of CD8^+^ T cells rather than NK and Treg cells in hPBMCs. hPBMCs were treated with 0.5 nM aldesleukin, αPD-L1 × WT IL-2, αPD-L1 × non-α IL-2v, or MB2033 in **a** resting or **b** activated condition in vitro, and total cell numbers were counted after 7 days. The results are representative of at least two independent experiments. Data are shown as the mean ± SEM of triplicates. Statistical analysis was performed using one-way ANOVA. ** and *** represent significant difference versus to vehicle, and ### represents significant difference between indicated groups (*p < 0.05, **p < 0.01 and ***, ###*p* < 0.001)

Unlike other constructs with high affinity for IL-2Rs, MB2033 did not induce the proliferation of CD8^+^ T, NK, or Treg cells under resting conditions (Fig. 4a). However, under activation conditions that mimic the tumor microenvironment, MB2033 effectively induced the proliferation of CD8^+^ T cells. Additionally, compared to the effect of αPD-L1 × WT IL-2, MB2033 showed a reduced tendency to induce Treg proliferation (Fig. 4b). Thus, we speculated that MB2033 does not induce the proliferation of immune cells in the resting state in the periphery, where no target tumor is present. However, MB2033 effectively induced the proliferation of effector CD8^+^ T cells only in the activated state within the tumor where multiple antigens were encountered.

### MB2033 enhances the cytotoxicity of immune cells to tumors

**Fig. 5:**
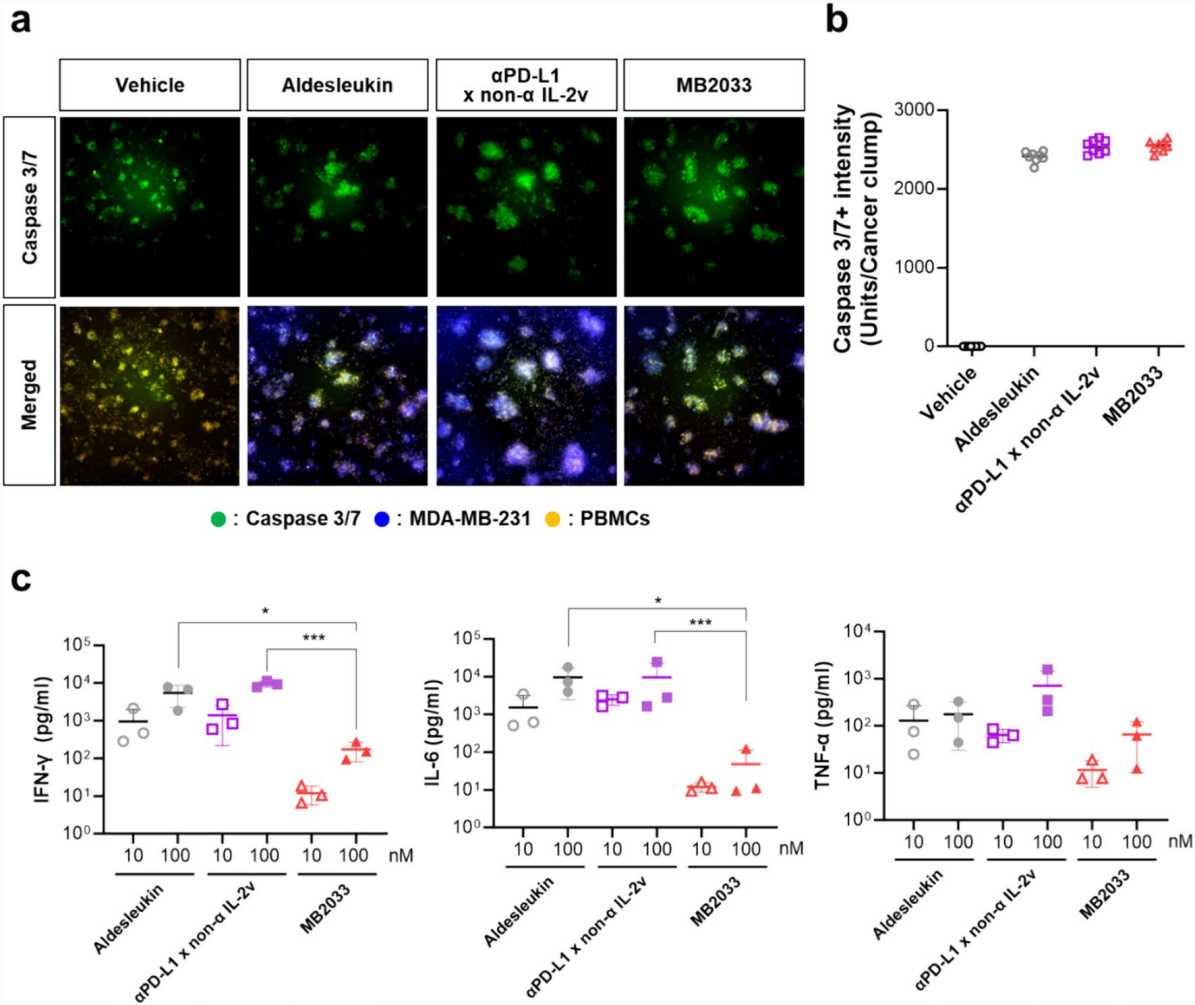
MB2033 enhanced the cytotoxicity effects of immune cells with less cytokine release than aldesleukin and αPD-L1 × non-α IL-2v. **a** To assess the anti-tumor effect of MB2033 in vitro, hPBMCs and MDA-MB-231 were co-cultured with aldesleukin, αPD-L1 × non-α IL-2v, or MB2033 at 10 nM for 48 h. The viability of tumor cells was measured by micro-confocal imaging at 20 × magnification. Live MDA-MB-231 cells were indicated by blue nuclei and hPBMCs, and caspase 3/7-mediated cell death was indicated by orange and green, respectively. Overlapping MDA-MB-231 (blue) and hPBMCs (orange) was assigned to the cancer clamp (white), and the caspase 3/7^+^ (green) intensity unit in the cancer clamp is shown in **b**. **c** To compare cytokine release under off-tumor conditions by test articles, hPBMCs were incubated with 10 or 100 nM of test articles without tumor cells. After 72 h, IFN-γ, IL-6, and TNF-α from the supernatant were measured using a cytometric bead array. The results were representative, and the data were collected from three individual PBMCs. Statistical analysis was performed using paired t-tests (*p < 0.05, **p < 0.01, ***p < 0.001)

To ascertain whether MB2033 not only induces the activation and proliferation of immune cells but also immune cell-mediated cytotoxic effects, hPBMCs and MDA-MB-231, as effector and target cells, respectively, were co-cultured with each drug, and the extent of tumor cell death was observed through fluorescence image analysis (Fig. 5a, b). In the vehicle group, despite interactions between MDA-MB-231 cells and hPBMCs, numerous clumps of cells formed by the aggregation of immune cells and MDA-MB-231 cells were observed; however, the caspase 3/7^+^ signal intensity within these clumps was very low, indicating low cytotoxicity. Conversely, in the aldesleukin-, αPD-L1 × non-α IL-2v-, and MB2033-treated groups, the clump size increased along with an increase in caspase 3/7^+^ intensity within the clumps, allowing for efficient tumor cell killing. Notably, there was no difference in cytotoxicity based on the affinity of each drug for IL-2Rs.

In the presence of target tumor cells in vitro, MB2033 induced abundant pro-inflammatory cytokine secretion, which was comparable to the levels induced by aldesleukin. However, in the off-tumor condition, the concentrations of IFN-γ, IL-6, and TNF-α released from MB2033-treated hPBMCs were lower (data not shown) than those released following the treatment of aldesleukin or αPD-L1 × non-α IL-2v (Fig. 5c). This suggests that MB2033 may not induce unnecessary cytokine release when exposed to the bloodstream in the absence of tumors.

### MB2033 shows anti-tumor efficacy without adverse effects in a mouse syngeneic model

**Fig. 6:**
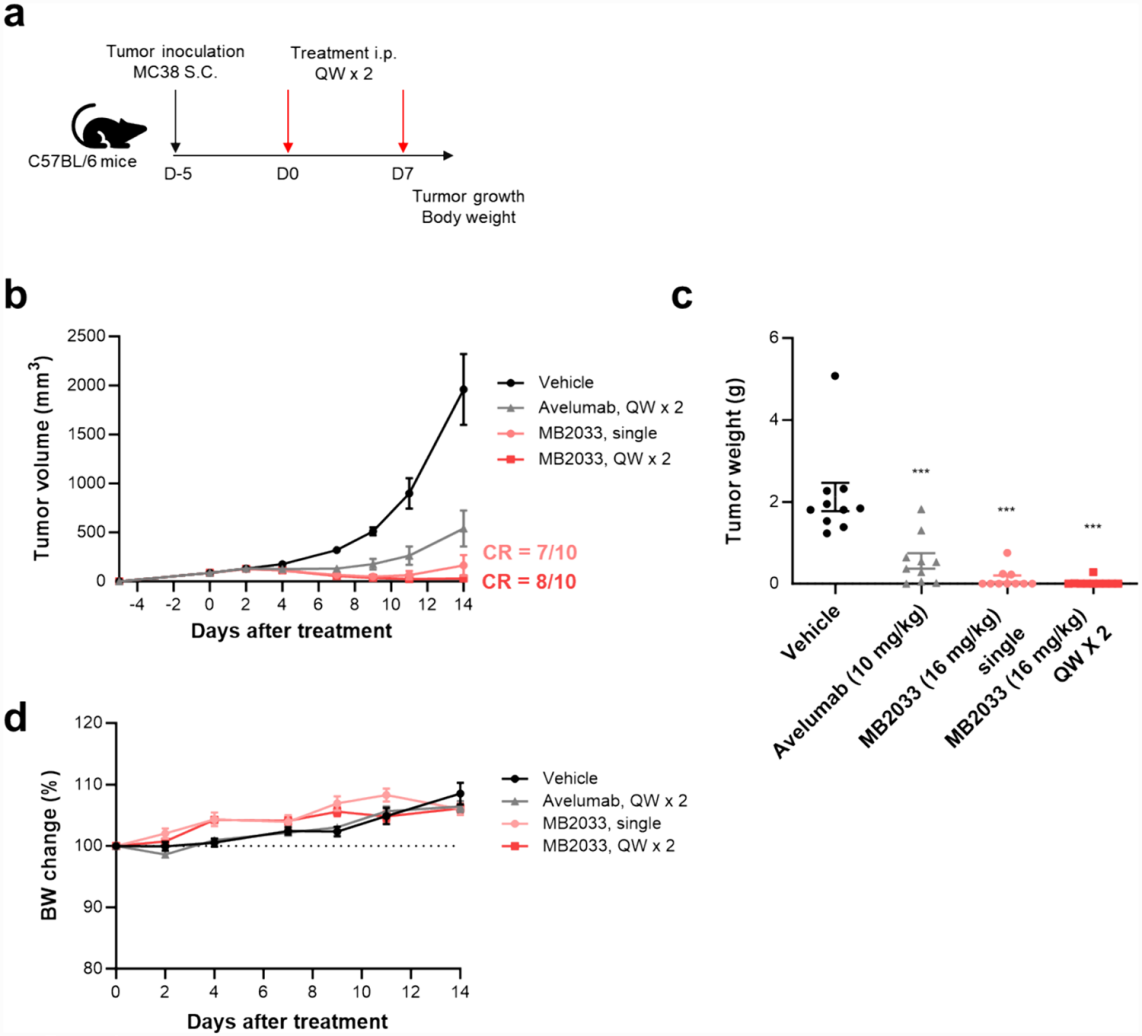
The anti-tumor efficacy of MB2033 was observed without body weight loss in an MC38 colon cancer model. **a** Experimental design for anti-tumor efficacy study. C57BL/6 mice were subcutaneously injected on day 0 with MC38 cells (1 × 10^6^ cells). 5 days after tumor inoculation, mice (*n* = 10/group) were randomly distributed to groups with an average tumor volume of ~ 90 mm^3^ per group. Avelumab (10 mg/kg) was intraperitoneally administered once a week for 2 weeks. MB2033 (16 mg/kg) was intraperitoneally administered as a single dose, or once a week for 2 weeks. **b** Tumor volumes were measured three times a week. **c** Average and individual tumor weight were measured at the end of the study. **d** Body weight changes compared with day 0. The dotted line represents day 0, which corresponds to 100%. Data are shown as the means ± SEMs of 10 animals. Statistical significance (***p < *0.001*) was calculated by one-way ANOVA followed by Tukey’s post-test

Consistent with the in vitro results, MB2033 effectively inhibited tumor growth in an MC38 colon cancer-bearing syngeneic mouse (Fig. 6a). Complete regression of tumors was observed in 7/10 mice with a single treatment and 8/10 mice treated once weekly twice (Fig. 6b, c). Importantly, the MB2033-treated group showed, no signs of body weight loss or clinical symptoms, a feature typically associated with conventional IL-2 drugs (Fig. 6d). Additionally, we compared the efficacy of MB2033 to that of avelumab in the B16F10 syngeneic model (*n* = 6/group), known for its highly aggressive, revealing a higher efficacy with 10 mg/kg MB2033 (tumor growth inhibition, TGI = 74.36%) compared to 5 mg/kg avelumab (TGI = 47.52%). Importantly, this study also confirmed the absence of vascular leakage syndrome (VLS), a critical adverse effect associated with IL-2-based drugs, as evidenced by the lack of observed increase in wet-pulmonary weight, a pathological indicator of VLS (Supplementary Fig. 1).

**Fig. 7:**
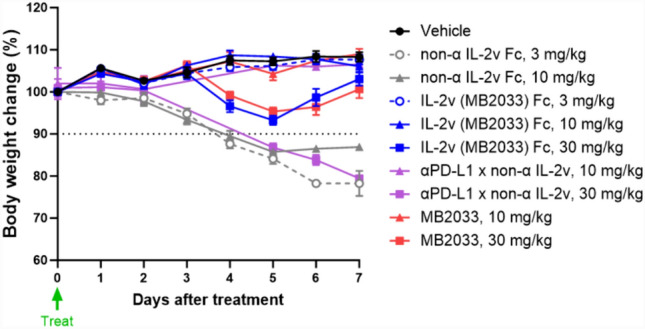
MB2033 induced less body weight loss than non-α IL-2v Fc monomer in normal C57BL/6 mice. Female C57BL/6 mice were intraperitoneally injected with non-α IL-2v Fc (3 and 10 mg/kg dose, *n* = 5), IL-2v (MB2033) Fc (3, 10, and 30 mg/kg dose, *n* = 5), αPD-L1 × non-α IL-2v (10 and 30 mg/kg dose, *n* = 3), or MB2033 (10 and 30 mg/kg dose, *n* = 5). Body weight and general clinical sign were observed daily. Data are shown as the means ± SEMs of five animals

In the toxicity assessment of normal C57BL/6 mice, 2/5 animals and 4/5 animals died on day 5 after administration of 3 and 10 mg/kg non-α IL-2v Fc, respectively. The surviving mice in the 3 mg/kg group also showed a body weight loss of approximately 21.8% on day 7 after treatment compared to that on day 0. Similarly, after treatment with αPD-L1 × non-α IL-2v at doses of 10 and 30 mg/kg, mortality was observed on day 5. However, in the IL-2v (MB2033)-Fc-treated group, there were no general clinical symptoms and change in body weight at the lower dose of 10 mg/kg, and temporary weight loss of only 6.7% with subsequent recovery for the higher dose of 30 mg/kg. Treatment with MB2033 cells including the hPD-L1 arm showed a similar tendency (Fig. 7). Therefore, MB2033 was tolerable up to a dose of 30 mg/kg and recoverable body weight loss following MB2033 administration was not due to the anti-hPD-L1 arm. Therefore, MB2033 appears to be a suitable immunotherapeutic agent for tumor killing with minimal side effects.

### Pharmacokinetics of MB2033

**Fig. 8:**
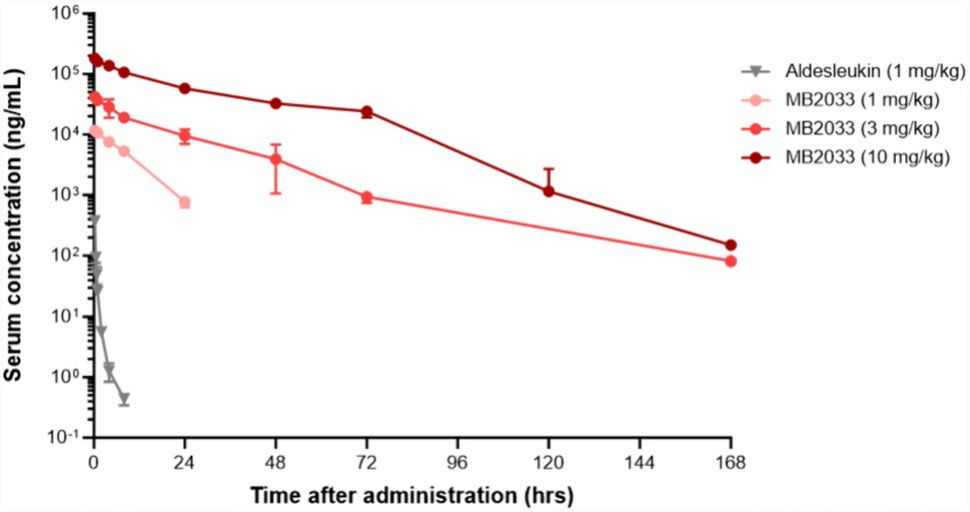
Pharmacokinetic profiles in normal ICR mice after single intravenous administration of MB2033

In a single-dose intravenous study in ICR mice, administration of MB2033 at 10 mg/kg demonstrated tolerability with no observed clinical signs or changes. Systemic exposure to MB2033 increased by greater than the dose proportional level between 1 and 10 mg/kg (area under the curve [AUC], 1 mg/kg: 117,986 ng·h/mL, 3 mg/kg: 650,308.3 ng·h/mL, and 10 mg/kg: 4,765,584.3 ng·h/mL). At a 1 mg/kg, the AUC of MB2033 increased by 725.2-fold compared with that of the reference compound aldesleukin (162.7 ng·h/mL), indicating an improvement in the in vivo stability and exposure to MB2033 compared with those of the approved IL-2 drug aldesleukin (Fig. 8).

Concentration–time profiles of MB2033 after single intravenous dose at 1, 3, and 10 mg/kg and aldesleukin after single intravenous dose at 1 mg/kg in male ICR mice (*n* = 3 animals/time point/group). Serum concentrations (mean ± SD) of aldesleukin and MB2033 were determined by ELISA.

## Discussion

To improve conventional cancer immunotherapies, we developed a novel αPD-L1 × IL-2v fusion protein, named MB2033. This fusion protein has the characteristic of tumor-specific targeting and blocking the PD-1/PD-L1 interaction through the αPD-L1 arm, while also preferentially enhancing the activation and proliferation of CD8^+^ T cells over Tregs due to the attenuated affinity of the novel IL-2v for IL-2Rβγ and lack of affinity for IL-2Rα, resulting in anti-tumor activity.

pSTAT5 and hPBMC expansion assays confirmed the limitations of WT IL-2 in terms of efficacy and systemic toxicity. Aldesleukin, containing WT IL-2, preferentially induces severe toxicities and the excessive expansion of Tregs that consistently express IL-2Rα [18]. Efforts have been made to overcome these limitations by developing engineered IL-2vs that reduce the binding to IL-2Rα on Tregs and endothelial cells [19, 20] or by increasing the affinity for IL-2Rβ on effector cells [21, 22]. We also observed that non-α IL-2v and non-α and βγ-attenuated IL-2v activated Tregs at levels similar or slightly higher than the activation of CD8^+^ T cells, which could potentially reduce immune-suppressive responses. As MB2033 can reduce the activation of immunosuppressive responses, the therapeutic efficacy may increase by activating CD8^+^ T cells, which was nearly equivalent to that induced by WT IL-2, as revealed by pSTAT5 assays. However, Treg cell activation by MB2033 was induced at levels 352-fold lower compared with WT (EC_50_; WT IL-2 = 0.012 nM, MB2033 = 4.135 nM). Therefore, MB2033 could potentially induce a more effective immune response in tumors than WT IL-2. Moreover, the selective immune cell activation by MB2033 initiates proliferation, indicating that MB2033 induces the proliferation of CD8^+^ T cells rather than that of Tregs.

Cancer immunotherapies based on anti-PD-1/PD-L1 have transformed the landscape of cancer treatment [23, 24]. Despite the ability of PD-1/PD-L1 blockade to alleviate the suppression of T cell responses, T cells exhibit incomplete functionality and restricted expansion within the tumor. Consequently, only a minority of patients experience complete responses [25]. We observed that MB2033 efficiently bound to tumor cells in an hPD-L1 expression-dependent manner. This suggests the potential for the selective delivery of IL-2v to the tumor cell environment. Accordingly, we anticipate that MB2033 could be an effective therapeutic agent to overcome the challenge of ICI resistance. This function was corroborated at the cellular level using an in vitro tumor cell lysis assay. The IL-2v targeting PD-L1 induced the formation of larger cancer apoptotic clumps than those formed with treatment of aldesleukin. Moreover, the caspase 3/7^+^ intensity, indicative of cancer clump death, was similar in all groups except the vehicle group. This suggests that MB2033, despite having intermediate affinity for IL-2Rs, can sufficiently activate effector immune cells to induce a tumor cell-killing effect. Furthermore, the ability of αPD-L1 × IL-2v to target tumor cells through αPD-L1 enables the delivery of IL-2v close to the tumor, which can also explain its effective tumor cell-killing effect despite the reduced affinity for IL-2Rs.

MB2033 exhibited superior anti-tumor efficacy compared to avelumab and aldesleukin in a PD-L1-highly expressed MC38 colon cancer mouse model [26]. This enhanced efficacy is likely attributed to the delivery of IL-2v through the anti-PD-L1 moiety, as evidenced by increased tumor cell killing when IL-2v is combined with anti-PD-L1 (Supplementary Fig. 3). Safety evaluations revealed no significant increase in lung weight induced by MB2033, even in the highly effective B16F10 model (Supplementary Fig. 1). Single administration of MB2033 up to 30 mg/kg was safe in normal mice, indicating its potential as a low-risk anti-cancer therapy. However, limitations include the lack of evidence for immune response within the tumor in vivo. Despite this, the in vitro results suggest that MB2033 mediates efficacy through selective proliferation of CD8^+^ T cells over Treg cells. Future studies focusing on MB2033’s impact on the tumor microenvironment from an immunological perspective may complement these findings.

MB2033 has a pharmacokinetic profile with an extended AUC compared with that of aldesleukin, suggesting that systemic exposure is likely adequate to maintain saturating drug levels in the tumor for several days. This represents a significant advance compared with the short half-life of aldesleukin and should facilitate less frequent dosing. Additionally, the observed half-life of MB2033 (16 h at 3 and 10 mg/kg) was comparable to that reported in mice for the monoclonal antibody avelumab (12–40 h) [27]. Therefore, MB2033 would be amenable to the general dosing regimen of immuno-oncology therapies (i.e., 2- or 3-week administration intervals).

The anti-PD-L1 × IL-2v fusion protein, conceived upon an innovative bispecific antibody platform, addresses the limitations of conventional IL-2 therapies by mitigating adverse effects while enhancing anti-tumor efficacy.

Our ongoing investigations envisage enhanced therapeutic efficacy in patients with refractory solid tumors. Moreover, the unique IL-2v technology presents a compelling avenue for developing novel immunotherapeutic modalities through synergistic combinations with diverse anticancer antibodies.

### Supplementary Information

Below is the link to the electronic supplementary material.

Supplementary file1 (PDF 621 KB)

## Data Availability

The datasets generated during and/or analyzed during the current study are available from the corresponding author on reasonable request.

## Abbreviations

EC_50_: Half-maximal effective concentration
ICIs: Immune checkpoint inhibitors
IL-2: Interleukin-2
IL-2v: IL-2 variant
*K*_*D*_: Binding affinity
PD-L1: Programmed death ligand 1
rhIL-2: Recombinant human IL-2
Tregs: Regulatory T cells
