# Supplementary material for: MB2033, an anti-PD-L1 × IL-2 variant fusion protein, demonstrates robust anti-tumor efficacy with minimal peripheral toxicity
Source: Cancer Immunol Immunother. 2024 Jun 4;73(8):157. doi: 10.1007/s00262-024-03742-1 (PMC11150458; doi:10.1007/s00262-024-03742-1)
Supplement: Supplementary file 1 — Supplementary file1 (PDF 621 KB) [file 262_2024_3742_MOESM1_ESM.pdf]

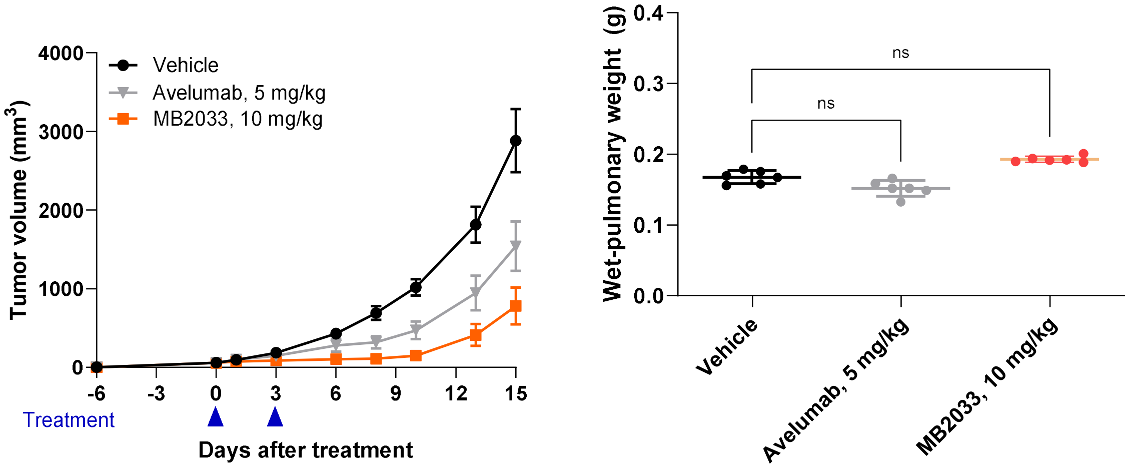

Supplementary Fig. 1 Anti-tumor efficacy by MB2033 administration in B16F10 syngeneic mouse model. C57BL/6 mice were subcutaneously injected on day 0 with B16F10 cells (1×10^6 cells/mouse). At 6 days after tumor inoculation, mice (n=6/group) were randomly distributed to groups with an average tumor volume of ~60 mm^3 per group. Test articles were intraperitoneally administered twice with a 3-day interval. Average and individual wet-pulmonary weight were measured at the end of the study. Data are presented as means±SEMs, and statistical significance was determined using one-way ANOVA followed by Dunnett's T3 multiple comparisons, *p<0.05*, p<0.005**, p<0.001****

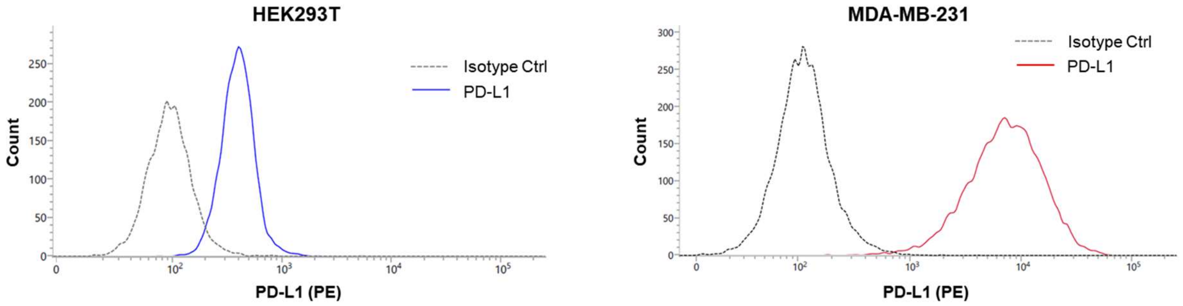

	PE-MFI
Isotype control	121
PD-L1	407

	PE-MFI
Isotype control	126
PD-L1	7,498

Supplementary Fig. 2 PD-L1 expression level in HEK293T and MDA-MB-231.

To establish criteria for PD-L1^{high} and PD-L1^{low} cells, HEK293T and MDA-MB-231 cells were stained with PE anti-PD-L1 antibody, followed by flow cytometry analysis to compare the mean fluorescence intensity (MFI) of PD-L1 to that of the isotype control. The expression levels of PD-L1 in each cell type can be observed in the provided histograms. The MFI measured for isotype or anti-PD-L1-PE is indicated in the table below in the histogram

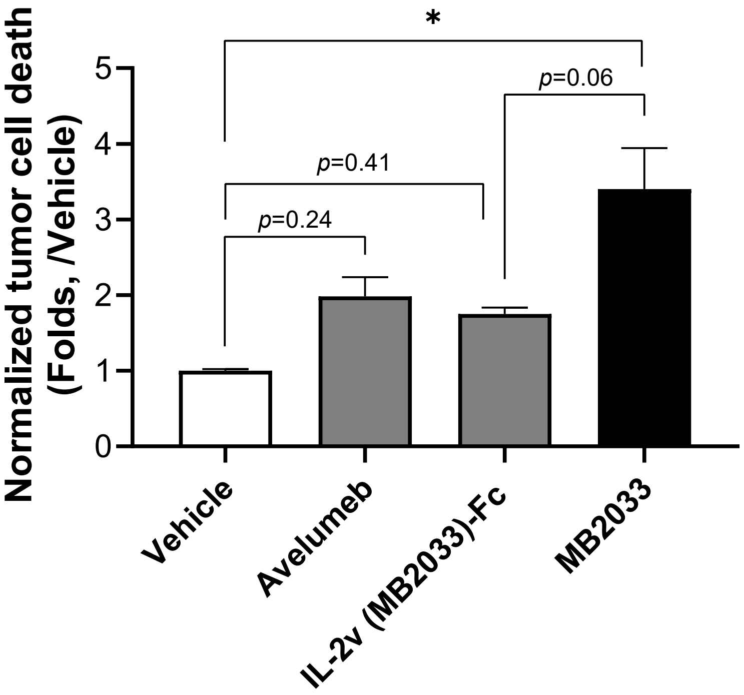

Supplementary Fig. 3 Tumor cell killing effect by the increased immune response induced by MB2033 *in vitro*

hPBMC and MDA-MB-231 (stained with CellTrace^{TM} Violet$^{)}$ were co-cultured with avelumab, IL-2v (MB2033)-Fc, or MB2033 at 10 nM for 48 hrs. Tumor cell death (CellTrace^{TM} Violet^{+}7AAD^{+} cells) was quantified using flow cytometry (n=3). Data are presented as means±SEMs, and statistical significance was determined using one-way ANOVA followed by Dunnett's T3 multiple comparisons, $p<0.05$*
